# A Requirement for Neutrophil Glycosaminoglycans in Chemokine:Receptor Interactions Is Revealed by the Streptococcal Protease SpyCEP

**DOI:** 10.4049/jimmunol.1801688

**Published:** 2019-04-22

**Authors:** Jennifer Goldblatt, Richard Ashley Lawrenson, Luke Muir, Saloni Dattani, Ashley Hoffland, Tomoko Tsuchiya, Shiro Kanegasaki, Shiranee Sriskandan, James E. Pease

**Affiliations:** * National Heart and Lung Institute, Imperial College London, London SW7 2AZ, United Kingdom;; † Department of Medicine, Imperial College London, London W12 0NN, United Kingdom;; ‡ Asthma U.K. Centre in Allergic Mechanisms of Asthma, London, United Kingdom; and; § Research Institute, National Center for Global Health and Medicine, Shinjuku-ku, Tokyo 162-8655, Japan

## Abstract

To evade the immune system, the lethal human pathogen *Streptococcus pyogenes* produces SpyCEP, an enzyme that cleaves the C-terminal α-helix of CXCL8, resulting in markedly impaired recruitment of neutrophils to sites of invasive infection. The basis for chemokine inactivation by SpyCEP is, however, poorly understood, as the core domain of CXCL8 known to interact with CXCL8 receptors is unaffected by enzymatic cleavage. We examined the in vitro migration of human neutrophils and observed that their ability to efficiently navigate a CXCL8 gradient was compromised following CXCL8 cleavage by SpyCEP. SpyCEP-mediated cleavage of CXCL8 also impaired CXCL8-induced migration of transfectants expressing the human chemokine receptors CXCR1 or CXCR2. Despite possessing an intact N terminus and preserved disulfide bonds, SpyCEP-cleaved CXCL8 had impaired binding to both CXCR1 and CXCR2, pointing to a requirement for the C-terminal α-helix. SpyCEP-cleaved CXCL8 had similarly impaired binding to the glycosaminoglycan heparin. Enzymatic removal of neutrophil glycosaminoglycans was observed to ablate neutrophil navigation of a CXCL8 gradient, whereas navigation of an fMLF gradient remained largely intact. We conclude, therefore, that SpyCEP cleavage of CXCL8 results in chemokine inactivation because of a requirement for glycosaminoglycan binding in productive chemokine:receptor interactions. This may inform strategies to inhibit the activity of SpyCEP, but may also influence future approaches to inhibit unwanted chemokine-induced inflammation.

## Key Points

- SpyCEP-cleaved CXCL8 is unable to bind and activate CXCL8 receptors.
- Neutrophil glycosaminoglycans are required for migration along a CXCL8 gradient.

## Introduction

Chemokines and their receptors form part of a complex network, noted for their roles in positioning leukocytes and other cells via the process of chemotaxis or directed migration (1, 2). Neutrophils play a prominent part in responses of the innate immune system and are guided to sites of microbial infection by members of the ELR^+^ subgroup of CXC chemokines, which contain a Glu-Leu-Arg motif at their N terminus. Chief among the ELR^+^ chemokines is CXCL8/IL-8, which interacts with two principal receptors on the neutrophil surface known as CXCR1 (3) and CXCR2 (4). CXCL8 is expressed and secreted by tissue macrophages and other cells, for example, epithelial cells following bacterial infection (5), and serves to recruit neutrophils from the circulation to deal with the invading pathogen. Mice deficient in CXCR2, the major receptor for murine ELR^+^ chemokines, such as KC and MIP-2, exhibit profound defects in neutrophil emigration to sites of both microbial (6) and sterile-induced inflammation (7), supporting the notion that gradients of ELR^+^ chemokines direct the chemotaxis of neutrophils in vivo. In a variety of inflammatory diseases, inadvertent or overexpression of CXCL8 has been associated with pathological consequences (8), and consequently, much effort has been put into the discovery of small molecule antagonists of CXCL8 receptors (9).

The importance of chemokines in coordinating leukocyte migration in host defense has not escaped the attention of microbes. Several pathogens have evolved ways to subvert the chemokine system and, hence, evade clearance by leukocytes. These include the synthesis of chemokine binding proteins by poxviruses, which neutralize the in vivo activity of chemokines (10, 11) and the production of enzymes by hookworms, which specifically degrade chemokines involved in eosinophil recruitment (12). *Streptococcus pyogenes* (group A *Streptococcus*; GAS) is known to cause a spectrum of infections, ranging from pharyngitis and impetigo to more invasive life-threatening diseases, such as necrotizing fasciitis, which is characterized by a marked and paradoxical paucity of neutrophil recruitment at sites of severe infection and heavy bacterial growth (13). Invasive *S. pyogenes* infection is associated with the upregulation of several genes encoding virulence factors; among which, the gene *cepA*/SpyCEP is of particular interest (14). The *cepA* gene encodes for a protein known as *S. pyogenes* cell envelope protease (SpyCEP) that specifically cleaves CXCL8 within the C-terminal α-helix, resulting in truncation of CXCL8 by 13 aa. The prominence of SpyCEP among GAS virulence factors has been ably demonstrated by loss-of-function and gain-of-function analyses (15, 16). SpyCEP-deficient strains have been found to be readily cleared in murine models of necrotizing fasciitis (15–17), whereas, conversely, heterologous expression of SpyCEP in *Lactococcus lactis* reproduced many of the features of severe necrotizing *S. pyogenes* infection in a hitherto avirulent bacterium, notably an inability to be cleared and an ability to disseminate to other organs (15). Immunity to SpyCEP confers additional protection in more virulent models of murine infection (18).

Of interest is the mechanism of action of SpyCEP, namely why C-terminal truncation of CXCL8 should result in a reduction of biological activity. Current models of chemokine:receptor activation conform to a two-step model in which, first, the chemokine receptor N terminus interacts with the chemokine core domain (chemokine recognition site 1; CRS1), tethering and orientating the chemokine so that, second, its N terminus can interact with the receptor ligand-binding pocket (chemokine recognition site 2; CRS2) (19). In agreement with this model, early structure/function studies of CXCL8 and the receptors CXCR1 and CXCR2 suggested major roles for the CXCL8 N terminus in receptor activation following ligand binding (20–22) and for the receptor N termini in ligand recognition (23, 24). In contrast, the C terminus of CXCL8 has been implicated in binding to glycosaminoglycans (GAGs) via a cluster of lysine residues (25, 26). Binding to GAGs on the surface of vascular endothelial cells allows chemokines to form stable or haptotactic gradients, which can be encountered by leukocytes rolling along the surface under the control of endothelial-expressed selectins (27). GAG binding was originally shown to be crucial for the in vivo activity of the chemokine CCL5. Mutant CCL5 molecules that are unable to bind to GAGs have been shown to retain the ability to induce leukocyte chemotaxis in vitro, but not leukocyte recruitment in vivo, when introduced into the peritoneum of mice (28).

Mutation of the CXCL8 C terminus to generate a CXCL8 species unable to bind GAGs has been shown in two separate studies to result in enhanced neutrophil recruitment when the chemokine is instilled into the lungs of mice, suggesting that GAGs modulate the spatiotemporal formation of CXCL8 gradients in vivo (26, 29). Supportive of this, the protein product of TNF-stimulated gene-6 (TSG-6) has been shown to inhibit CXCL8-induced neutrophil migration by binding to the C terminus of CXCL8, thereby inhibiting GAG binding (30). Similarly, neutrophil recruitment to the joints of mice following CXCL8 injection was significantly impaired by i.v. administration of a peptide derived from the C terminus of CXCL9, which has been shown to compete with chemokines for binding to GAGs (31).

Although reduced interaction with endothelial GAGs may explain some of the effects of SpyCEP observed in vivo, it cannot explain the wider effects of SpyCEP on neutrophils that are evident in vitro, such as reduced CD62L shedding or chemotaxis (13). If N-terminal–mediated receptor binding of SpyCEP-truncated CXCL8 were preserved, and this were independent of GAG binding, some CXCR1 and CXCR2 function might be expected to be preserved. In this study, we examined the effects of SpyCEP cleavage upon neutrophil migration and the interaction of CXCL8 with the receptors CXCR1 and CXCR2 and with cell surface GAGs. We used a combination of cell transfectants and freshly isolated human neutrophils to examine different aspects of these processes. We highlight a previously unappreciated role for the C-terminal α-helix of CXCL8 in chemokine receptor binding and signaling, which is explained by a requirement for GAG binding. We suggest that the impaired recruitment of neutrophils during severe *S. pyogenes* infections results from not only an inability to generate a transendothelial chemokine gradient, but also an inability of neutrophils to respond to chemokines that are cleaved by SpyCEP.

## Materials and Methods

### Materials

All materials were obtained from Thermo Fisher Scientific (Renfrew, U.K.) unless otherwise stated. Recombinant human CXCL8 was obtained from Bio-Techne (Abingdon, U.K.) and was purchased in both the 72 aa (CXCL8^1–72^) and 77 aa (CXCL8^1–77^) forms. All experiments were performed with the 72 aa form of CXCL8 unless stated otherwise. Other recombinant chemokines were from PeproTech (London, U.K.). fMLF was from Bio-Techne. The glycanase mixture of heparinase II, heparinase III, and chondroitinase ABC was purchased from Sigma-Aldrich (Poole, U.K.).

### Cell culture and transfection

The mouse pre–B cell line L1.2 was maintained and transfected with plasmids by electroporation as previously described (32). The plasmid vector pcDNA3 encoding HA-tagged variants of human CXCR1 and CXCR2 were purchased from the cDNA Resource Center (Bloomsburg University, PA). Four hours following transfection, cultures were supplemented with sodium butyrate (Sigma-Aldrich) at a final concentration of 10 mM. Overnight culture in the presence of sodium butyrate enhances the transient expression of chemokine receptors in this system. Expression of HA-tagged CXCR1 or CXCR2 was confirmed prior to experimentation (data not shown) by the use of an anti-HA monoclonal and flow cytometry analysis by FACSCalibur (BD Biosciences, Oxford, U.K.) as previously described (32).

Human neutrophils were isolated from whole blood obtained from a subcollection of the Imperial College Tissue Bank, taken from informed, consenting, healthy normal subjects. Neutrophils were freshly isolated by negative selection using the MACSxpress Neutrophil Isolation Kit according to the manufacturer’s instructions (Miltenyi Biotec, Woking, U.K.), followed by a single RBC lysis step (using hypo/hypertonic solutions).

### Chemokine cleavage by SpyCEP

*Emm*81 *S. pyogenes* strain H292 has been previously defined as a high SpyCEP-producing strain (13, 33) and was used as a source of SpyCEP with a molecular mass of ∼160 kDa. H292 was grown for 16 h at 37°C in an atmosphere of 5% CO_2_ in RPMI 1640 (Thermo Fisher Scientific) and the supernatant retained. Culture supernatants were centrifuged at 2500 × *g* for 10 min at 4°C to pellet bacteria; following which, they were passed through a 0.2-μm filter (VWR, Lutterworth, U.K.) and split into aliquots stored at −20°C. GAS strain H575 is an isogenic mutant of H292 in which the majority of *cepA* has been deleted, leading to production of an inactive, truncated N-terminal SpyCEP fragment of ∼40 kDa (15). Supernatants from this strain were produced in an identical fashion as a negative control for SpyCEP cleavage of chemokines. Chemokine cleavage assays were carried out by digesting a known amount of recombinant CXCL8 at 37°C for 16 h in supernatant from the H292 strain (+SpyCEP) or H575 (−SpyCEP) GAS strains; after which, it was diluted in assay buffer (RPMI 1640 + 0.1/% BSA) to give the required concentration of chemokine.

### Chemotaxis assays

Dilutions of CXCL8 treated with either the H575 or H292 supernatants were made in assay buffer (RPMI 1640 + 0.1/% BSA). Migration toward CXCL8 was assessed using L1.2 cell transfectants expressing HA-CXCR1 or HA-CXCR2 in modified Boyden chamber assays, as previously described (32), using ChemoTx chambers with a 5-μm pore size (Neuro Probe, Gaithersburg, MD).

For the real-time analysis of migrating neutrophils, a 12-channel TAXIScan was employed (34) and used according to the manufacturer’s protocol (Effector Cell Institute, Tokyo. Japan). One microliter of a suspension containing 5 × 10^5^ neutrophils/ml was loaded into each chamber, and following alignment of the cells at one end of the terrace, 1 μl of 100 nM CXCL8 or 100 nM fMLF was added to the opposing end of the terrace (260 μm away), and cells were allowed to migrate along the ensuing chemoattractant gradient for 1 h at room temperature. Sequential image data were captured every minute as individual JPEGs, which were subsequently processed with ImageJ (National Institutes of Health), equipped with the manual tracking (Fabrice Cordelieres, Institut Curie, Orsay, France) and chemotaxis tool plugins (ibidi, Martinsried, Germany).

Individual experiments consisted of duplicate conditions for each chemokine, and data illustrated are collated from an equal number of experiments, as highlighted in the legends for Figs. 2, 3, and 6. The total numbers of cells tracked under each condition are shown in the top right-hand corner of each plot. For each individual cell, a variety of parameters were generated via the chemotaxis tool plugin, namely the accumulated distance traveled by each cell, its velocity, its directionality, and its forward migration index parallel to the gradient (FMI^‖^). Directionality is defined as the ratio of Euclidian distance:accumulated distance traveled. A value of 1 represents migration in a perfectly straight line. The FMI^‖^ is defined as the distance traveled by the cell in the *y*-axis (i.e., along the chemokine gradient) divided by the accumulated distance it traveled (35).

In experiments employing a glycanase mixture of heparinase I, heparinase II, and chondroitinase ABC, neutrophils were incubated with agitation with one unit per milliliter of enzyme for 30 min at 37°C, as previously described (36), before being washed once in phosphate-buffered saline prior to their immediate use in TAXIScan assays.

### Ligand-binding assays

Competitive binding assays were carried out with L1.2 cells expressing HA-CXCR1 or HA-CXCR2 using a previously described protocol (32). [^125^I]-CXCL8 was used as a radiolabel and was purchased from PerkinElmer Life Sciences (Boston, MA). Competition was with increasing concentrations of unlabeled CXCL8 incubated with either H292 or H575 culture supernatants and subsequently treated with Pefabloc SC (Roche, Lewes, U.K.), a serine protease inhibitor which inactivates SpyCEP (13). This was a precaution against digestion of the [^125^I]-CXCL8 tracer.

### Statistical analysis

Statistical analyses were carried out using Prism 6 (GraphPad Software, La Jolla, CA), and the tests are noted in the legends for Figs. 2–6. *p < 0.05, **p < 0.01, ***p < 0.001, and ****p < 0.0001.

## Results

### SpyCEP has broad spectrum activity for ELR^+^ CXC chemokines

**FIGURE 1.**
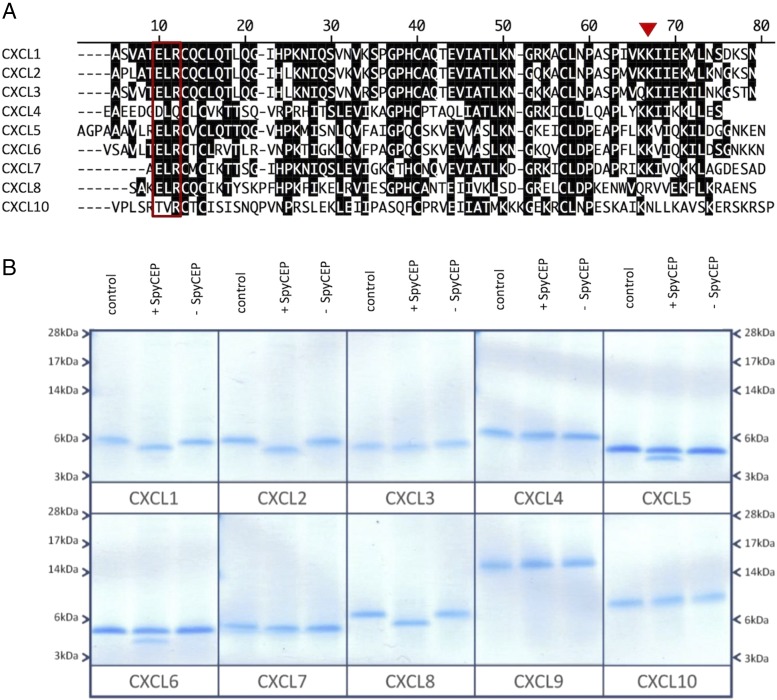
SpyCEP has broad spectrum activity for ELR^+^ CXC chemokines. (**A**) shows an alignment of a selection of mature human CXC chemokine sequences. The ELR motif shared by several of the chemokines is boxed and the previously determined site of cleavage of CXCL8 by SpyCEP is denoted by a red triangle (13). (**B**) shows the effects of SpyCEP cleavage on chemokine. Five hundred nanograms of chemokine was incubated with 1 μl of washed wild-type GAS cells (H292) or an isogenic *cepA* mutant (H575). Cleavage was allowed to proceed for 18 h at 37°C; after which, the proteins were separated by SDS-PAGE and stained with colloidal blue (B).

To ascertain the specificity of SpyCEP for neutrophil-recruiting CXC chemokines, a selection of recombinant chemokines with various degrees of homology (Fig. 1A) were incubated overnight with washed wild-type *S. pyogenes* cells (H292) (33) or the isogenic *cepA* mutant (H575) (15). Incubation with wild-type *S. pyogenes* cells evidently resulted in the cleavage of all ELR^+^ CXC chemokines as deduced by SDS-PAGE analysis (Fig. 1B), with a notable reduction in their m.w. In contrast, the non-ELR^+^ chemokines included as controls remained intact (CXCL4, CXCL9, and CXCL10). Incubation with control, SpyCEP-negative (H575) cells resulted in no detectable cleavage of any of the chemokines examined.

### Truncation of CXCL8 by SpyCEP results in impaired navigation of a chemokine gradient

**FIGURE 2.**
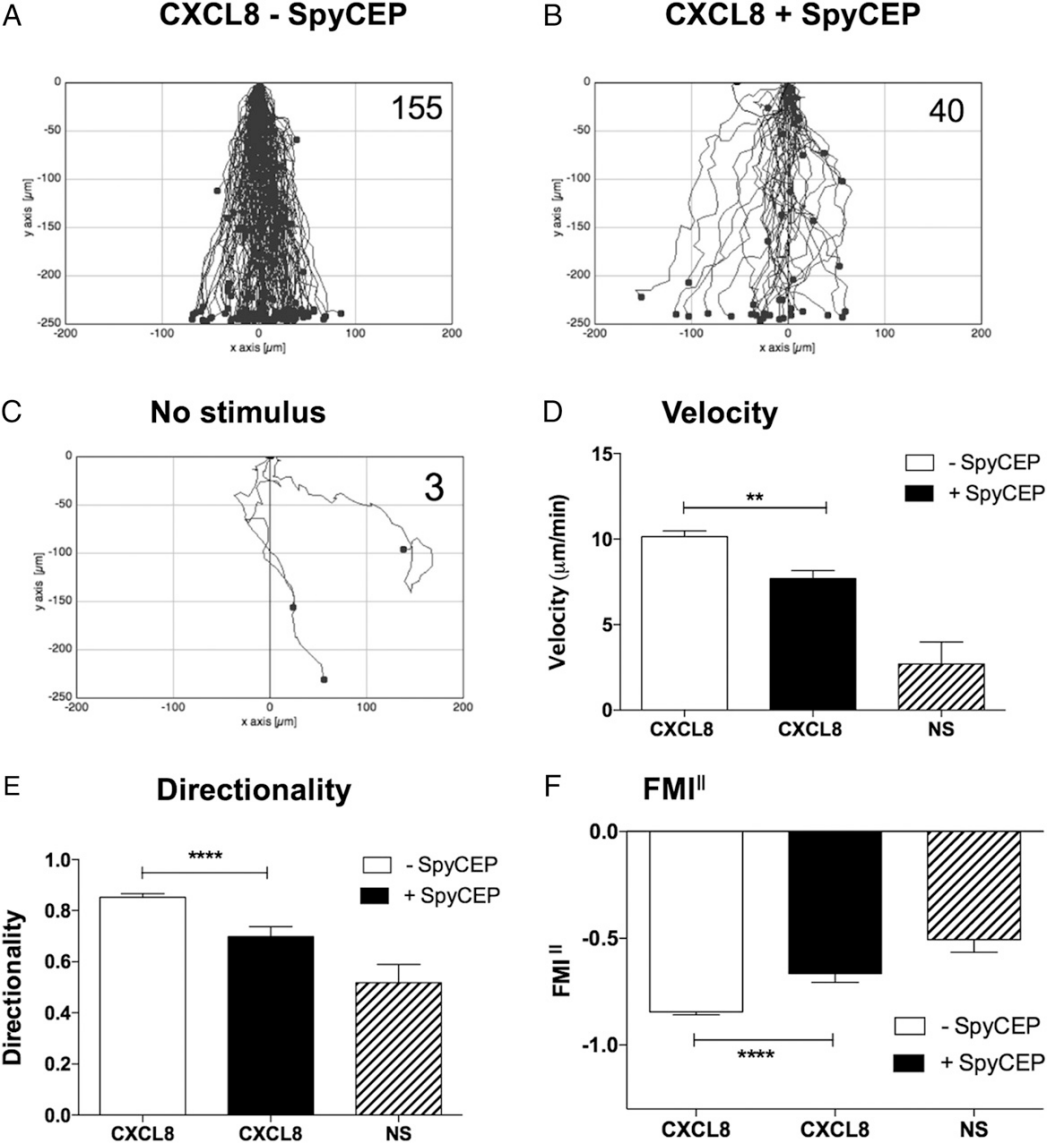
SpyCEP cleavage of CXCL8 results in significantly impaired migration of neutrophils. (**A**)–(**C**) show the collated tracks of individual migrating neutrophils (duplicate conditions) pooled from three independent experiments using different donors. Gradients of H575- or H292-treated CXCL8 were established in (A) and (B), respectively, whereas (C) shows the lack of neutrophil migration in the absence of chemokine. The total number of tracked cells from all three experiments is shown in the top right-hand corner. (**D**)–(**F**) show significant differences in velocity, directionality, and FMI^‖^ of the data in (A)–(C) following tracking analysis. Error bars represent the SEM. Statistical significances between CXCL8 treatments were determined by one-way ANOVA with Tukey posttest. **p < 0.01, ****p < 0.0001.

**FIGURE 3.**
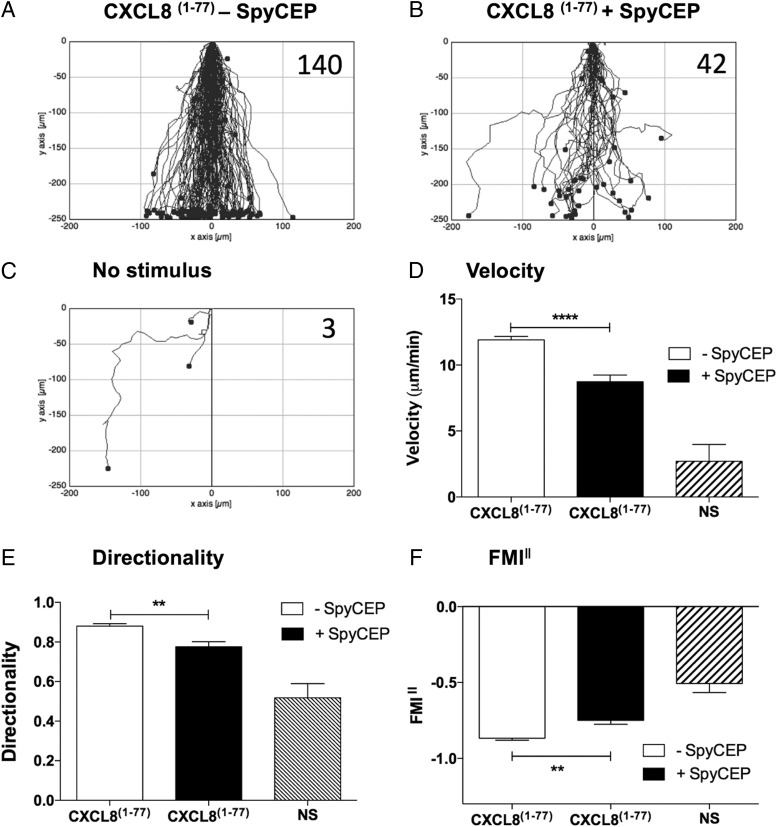
SpyCEP cleavage of the extended form of CXCL8 (CXCL8^1–77^) results in significantly impaired migration of neutrophils. (**A**)–(**C**) show the collated tracks of individual migrating neutrophils (duplicate conditions) pooled from three independent experiments using different donors. Gradients of H575- or H292-treated CXCL8^1–77^ were established in (A) and (B), respectively, whereas (C) shows the lack of neutrophil migration in the absence of chemokine. The total number of tracked cells from all three experiments is shown in the top right-hand corner. (**D**)–(**F**) show significant differences in velocity, directionality, and FMI^‖^ of the data in (A)–(C) following tracking analysis. Error bars represent the SEM. Statistical significances between CXCL8^1–77^ treatments were determined by one-way ANOVA with Tukey posttest. **p < 0.01, ****p < 0.0001.

The TAXIScan instrument was employed to assess the real-time migration of freshly isolated neutrophils. Initial studies directed at finding the optimum CXCL8 gradient showed that the addition of 1 μl of 100 nM CXCL8 to the chemotaxis chip resulted in robust migration (Supplemental Fig. 1). This concentration of CXCL8 was therefore used for subsequent studies. Intact CXCL8 was seen to elicit rapid neutrophil migration over a 1-h period of observation, with the majority of cells traversing the terrace in under 30 min in a direct fashion (Fig. 2A, Supplemental Video 1). SpyCEP-cleaved CXCL8 induced the migration of far fewer neutrophils, which lacked focus and appeared hesitant, making more turns and correspondingly taking much longer to traverse the terrace (Fig. 2B, Supplemental Video 2). Basal neutrophil migration in the absence of stimulus was minimal (Fig. 2C). Our observations were confirmed by single-cell tracking analysis with SpyCEP-cleaved CXCL8 inducing significantly slower migration than intact CXCL8 (Fig. 2D), accompanied by a significantly lower directionality component (Fig. 2E). When the forward migration indices parallel to the chemokine gradient were calculated (FMI^‖^), SpyCEP cleavage of CXCL8 was also seen to significantly impair the extent of migration compared with intact CXCL8 (Fig. 2F). Thus, SpyCEP cleavage of CXCL8 significantly impairs migration along the chemokine gradient, resulting in slower, less-directed neutrophil responses. When the N-terminally extended form of CXCL8 (CXCL8^1–77^) expressed by endothelial cells was employed in identical experiments, similar data were generated, with SpyCEP cleavage of CXCL8^1–77^ again observed to significantly impair chemotactic activity (Fig. 3A–F). Thus, SpyCEP is able to cleave both biologically active forms of CXCL8, rendering them less efficacious in terms of leukocyte recruitment.

### Truncation of CXCL8 by SpyCEP results in reduced activation and binding to CXCR1 and CXCR2

Previous reports have detailed the effects of CXCL8 truncation by SpyCEP on neutrophil function (13), but have not determined the relative contributions of CXCR1 and CXCR2 signaling. We set out to address these using constructs encoding N-terminal, HA-tagged variants of CXCR1 and CXCR2. These were transiently expressed at high levels in the mouse pre–B cell L1.2 according to published protocols (32), allowing ligand-binding assays and chemotaxis assays to be performed.

**FIGURE 4.**
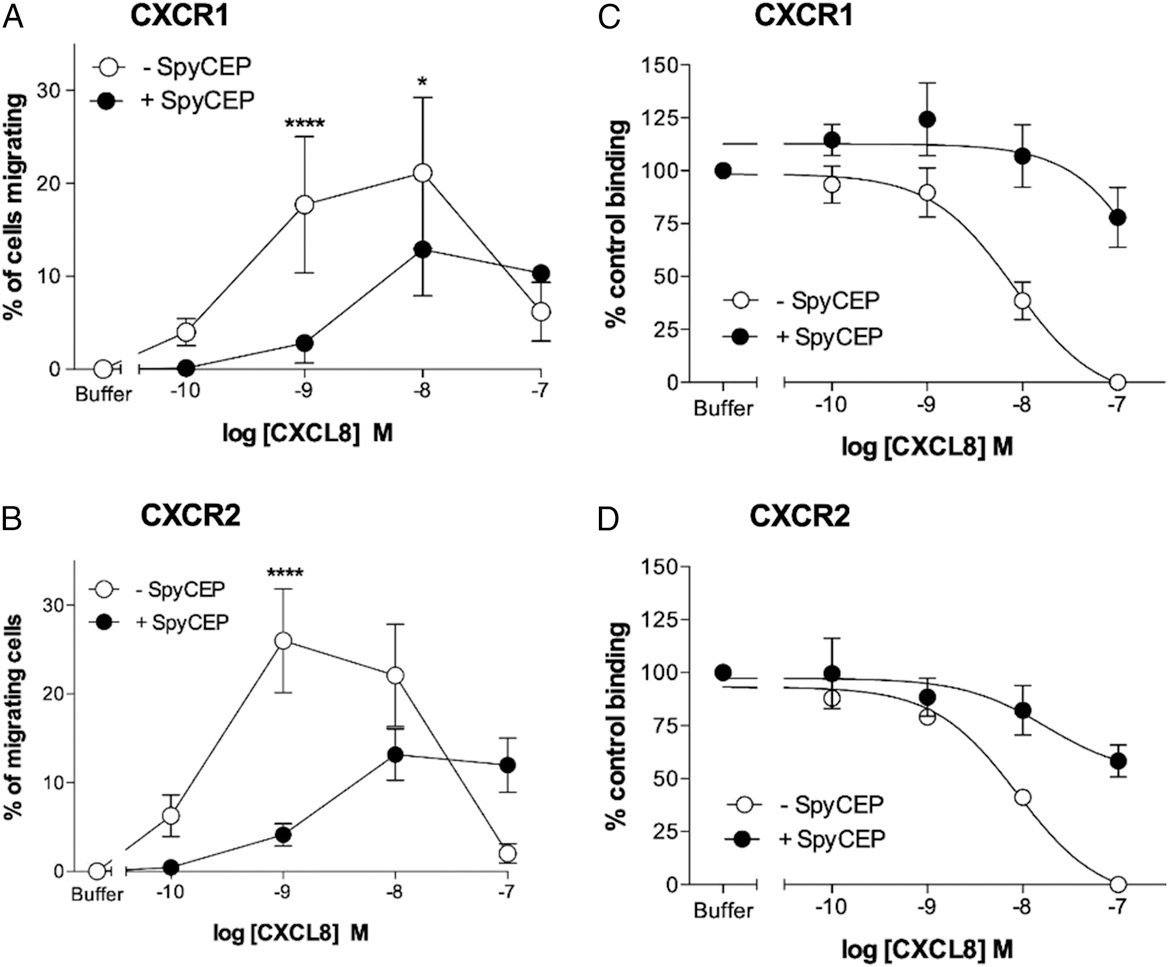
SpyCEP cleavage of CXCL8 inhibits binding and activation of CXCR1 and CXCR2. (**A**) and (**B**) show the ability of CXCL8 to compete for the binding of [^125^I]-CXCL8 to CXCR1 or CXCR2 transfectants following incubation with supernatants from either the H292 or H575 GAS strains (*n* = 6). (**C**) and (**D**) show the activities of identically treated CXCL8 in inducing the migration of CXCR1 or CXCR2 transfectants (*n* = 6). Error bars represent the mean ± SEM. *p < 0.05, ****p < 0.0001.

We first compared the ability of either SpyCEP-cleaved or intact CXCL8 to induce migration of CXCR1 and CXCR2 transfectants in modified Boyden Chamber assays (Fig. 4). Intact CXCL8 was efficacious in recruiting both CXCR1 and CXCR2 transfectants, with optimal migration seen in the 1–10 nM concentration range (Fig. 4A, 4B). In contrast, cleavage of CXCL8 with SpyCEP resulted in a significant reduction in both potency and efficacy compared with intact CXCL8. Previous mass spectrometry analysis confirms that the N-terminal domain and disulfide bridges within CXCL8 remain intact following SpyCEP cleavage (13), retaining the chemokine fold required for receptor binding. However, the reduced ability of SpyCEP-cleaved CXCL8 to provide a chemotactic gradient for neutrophils or for transfectants raised the possibility that cleaved CXCL8 might be unable to bind to its cognate receptors CXCR1 and CXCR2. Cleaved or uncleaved CXCL8 samples were diluted in assay buffer, and activity in competitive binding assays was evaluated using CXCR1 and CXCR2 transfectants. As expected, intact CXCL8 that had been incubated with the control (H575) supernatant readily displaced [^125^I]-CXCL8 from both CXCR1 and CXCR2 transfectants at nanomolar concentrations (IC_50_ values of 8.1 and 8.7 nM at CXCR1 and CXCR2, respectively; Fig 4C, 4D). In contrast, cleaved CXCL8 that had been incubated with SpyCEP was unable to displace 50% of the [^125^I]-CXCL8 from either receptor, even when used at a 1000-fold greater concentration than the labeled ligand, consistent with the reduced ability to signal through CXCR1 or CXCR2 observed in the migration assays. Thus, we conclude that it is the loss of the terminal 13 residues alone that adversely affects the binding of CXCL8 to both CXCR1 and CXCR2 and subsequent receptor activation.

### Truncation of CXCL8 by SpyCEP results in reduced GAG binding

**FIGURE 5.**
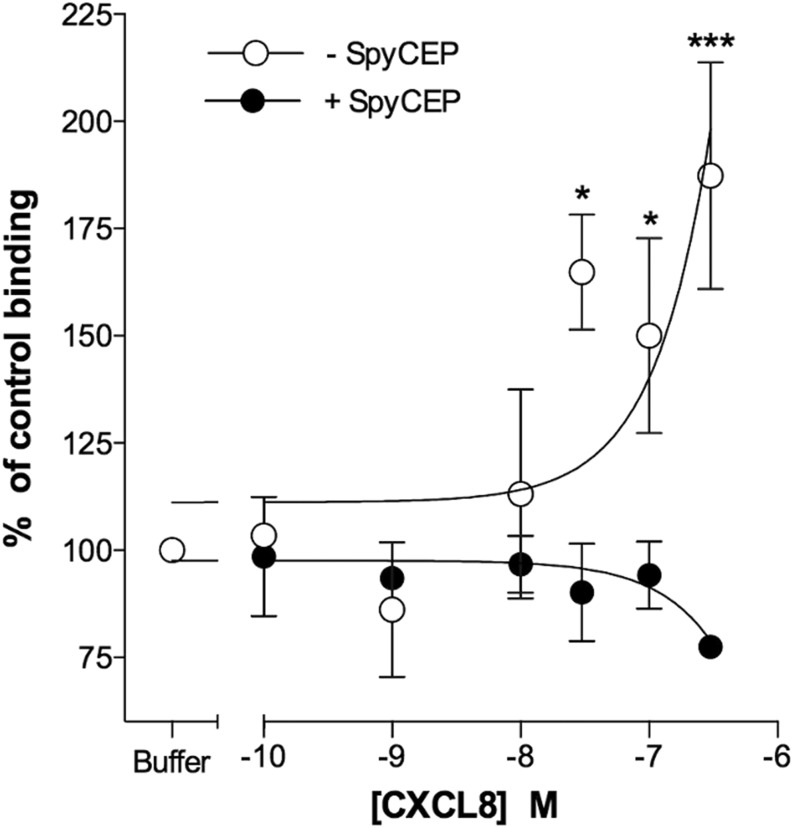
SpyCEP cleavage of CXCL8 ablates binding to heparin. Graph shows the relative abilities of H292- and H575-treated CXCL8 in binding to heparin Sepharose beads. Error bars represent the mean ± SEM (*n* = 6). Statistical significance between CXCL8 treatments was determined by two-way ANOVA with Sidak posttest. *p < 0.05, ***p < 0.001.

Chemokines have been shown to adhere with micromolar affinity to GAGs, which is essential for chemokine presentation on endothelial cells and the recruitment of leukocytes into tissues in vivo (37). The GAG binding domain of CXCL8 was shown by Kuschert et al. (25) to be comprised of five basic amino acids, namely K20 (within a region known as the proximal loop) and R60, K64, K67, and R68 in the C-terminal α-helix. Because truncation by SpyCEP at Q59 would remove four of these residues, we hypothesized that it may have deleterious effects on GAG binding. We therefore examined the ability of CXCL8 to form oligomers on heparin Sepharose beads following treatment with SpyCEP containing supernatants. In agreement with previous data from Hoogewerf and coworkers (38), increasing concentrations of intact CXCL8 lead to a corresponding increase in the proportion of CXCL8 bound to the beads (Fig. 5). In contrast, SpyCEP-cleaved CXCL8 was without activity in this assay, suggesting that removal of a large proportion of the GAG binding site by SpyCEP (four out of five basic residues) renders CXCL8 unable to bind to heparin.

### Effective navigation of a CXCL8 gradient requires neutrophil GAGs

**FIGURE 6.**
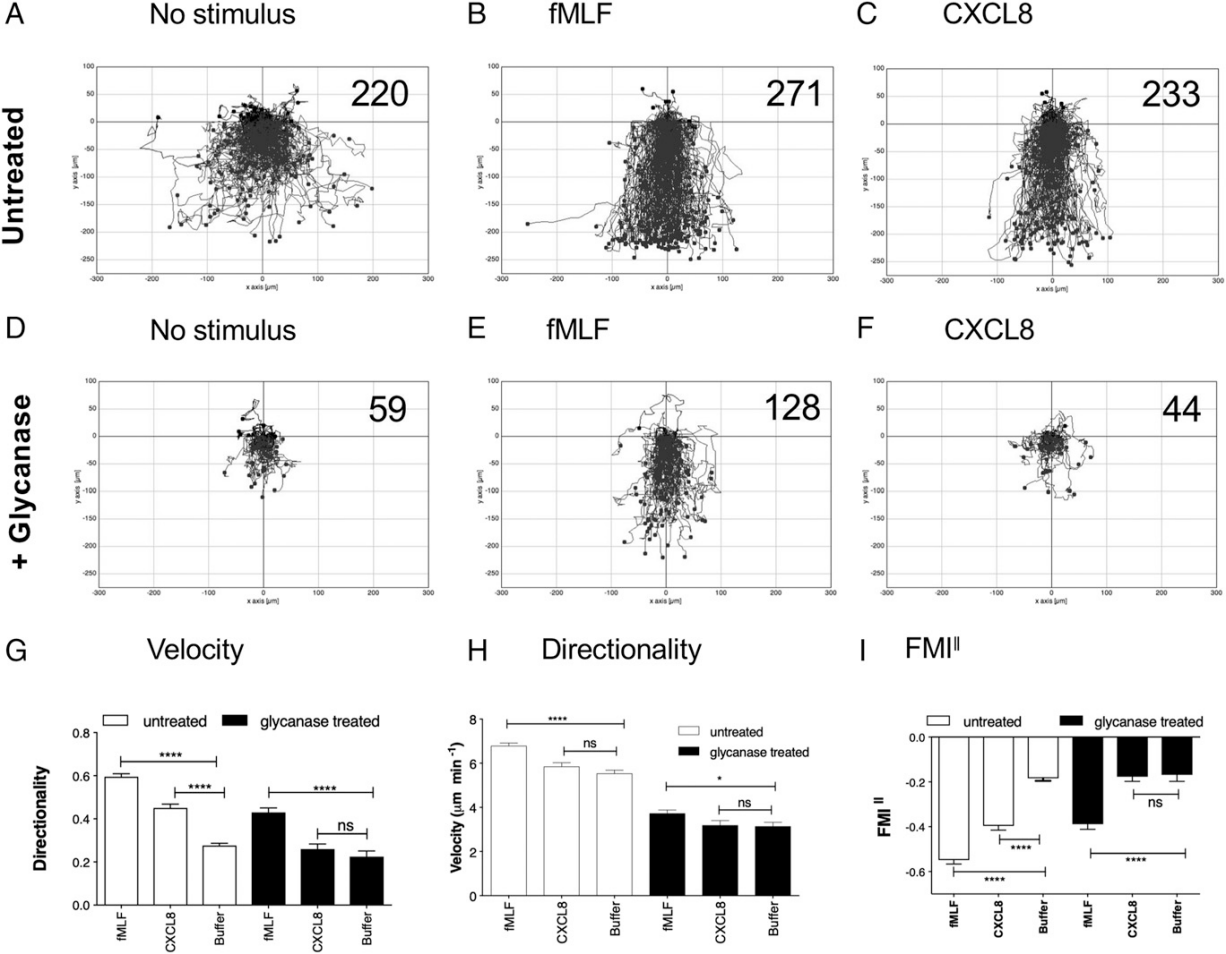
Removal of neutrophil GAGs specifically ablates migration along a CXCL8 gradient. (**A**)–(**F**) show the collated tracks of individual migrating neutrophils (duplicate conditions) pooled from three independent experiments using different donors. In (A)–(C), neutrophils were incubated in buffer alone prior to migration, whereas in (D)–(F), cells were treated with a glycanase mixture. Gradients of fMLF, CXCL8 were established where noted, with the total number of tracked cells shown in the top right-hand corner. (**G**)–(**I**) show significant differences in velocity, directionality, and FMI^‖^ of the data in (A)–(C) following tracking analysis. Error bars represent the SEM. Statistical significances between conditions were determined by one-way ANOVA with Tukey posttest. *p < 0.05, ****p < 0.0001.

Because SpyCEP cleavage impaired both GAG binding and neutrophil recruitment in vitro, we postulated that binding to GAGs on the neutrophil surface is a key step in the productive activation of neutrophil CXCL8 receptors. To test this postulate, we incubated neutrophils at 37°C in a glycanase mixture containing heparanases and chondroitinase, because both heparan sulfate and chondroitin sulfate–decorated GAGs have been shown to bind CXCL8 in vitro (39). After incubation, neutrophils were washed once in buffer, then assessed via TAXIScan for their ability to migrate along gradients of the tripeptide chemoattractant fMLF (deemed too small and uncharged to effectively bind to GAGs) or gradients of intact CXCL8. Incubation of neutrophils in buffer alone resulted in elevated basal migration, which appeared to be without direction in the absence of a stimulus (Fig. 6A).

The introduction of gradients of fMLF or CXCL8 induced obvious directional migration (Fig. 6B, 6C). Glycanase treatment resulted in an evident reduction in basal migration (Fig. 6D), although responses to fMLF remained intact (Fig. 6E). In contrast, migratory responses to CXCL8 were abolished by glycanase treatment (Fig. 6F). These findings were corroborated by single-cell tracking analysis. Glycanase treatment of neutrophils reduced the velocity and directionality of the CXCL8 responses so far, as they were indistinguishable from those seen in the absence of a stimulus (Fig. 6G, 6H). In contrast, despite glycanase treatment, fMLF-induced neutrophil migration remained significantly faster and more directional than that seen in the absence of a stimulus. Analysis of the FMI^‖^ indices further clarified these findings, with glycanase treatment of neutrophils seen to ablate migration along the CXCL8 gradient, whereas migration along the fMLF gradient remained significantly greater than that seen in the absence of a stimulus (Fig. 6I).

One potential explanation for the reduction in CXCL8 responses following GAG removal, is the possibility that the increased concentrations of soluble CXCL8 drive CXCR1/CXCR2 desensitization. If this is the case, then chemotactic responses to suboptimal gradients of CXCL8 would be envisaged to be improved by glycanase treatment of neutrophils. We examined this possibility, using gradients generated by the addition of 1 μl of 10 nM CXCL8 or 1 nM CXCL8 into the chemotaxis chamber (Supplemental Fig. 2). Untreated neutrophils were able to navigate the 10 nM CXCL8 gradient (Supplemental Fig. 2A) with significantly increased directionality and FMI^‖^ above the basal migration seen with no stimulus (Supplemental Fig. 2G, 2I). When presented with a 1 nM CXCL8 gradient, the migration was much less robust (Supplemental Fig. 2B), with analysis of the directionality and FMI^‖^ parameters showing them to be indistinct from those of basal migration (Supplemental Fig. 2G, 2I). Glycanase treatment of neutrophils was unable to improve neutrophil navigation of either the 10 nM CXCL8 or 1 nM CXCL8 gradients (Supplemental Fig. 2D, 2E), with migration along either gradient indistinct from basal migration (Supplemental Fig. 2G, 2I). Thus, we conclude that effective navigation of a CXCL8 gradient requires intact GAGs on the neutrophil cell surface, providing a potential explanation for the inactivating effects of C-terminal cleavage of CXCL8 by SpyCEP.

## Discussion

The paucity of neutrophils in severe necrotizing *S. pyogenes* infection has been directly attributed to the activity of SpyCEP, expression of which is upregulated in invasive isolates (15, 33). It was previously hypothesized that the cleavage of the CXCL8 C-terminal α-helix by SpyCEP resulted in abrogation of transendothelial chemokine gradients through the inability of cleaved CXCL8 to translocate to the luminal endothelial surface (13, 40). Although this may indeed be important, it cannot explain the inactivation of CXCL8 that we, and others, have observed in in vitro assays with neutrophils. In this study, we have demonstrated that SpyCEP cleavage of CXCL8 renders the chemokine unable to bind productively to its cognate receptors, providing an explanation for the observed inactivation. The broad activity of SpyCEP for a range of ELR^+^ CXC chemokines, coupled with the ability of *S. pyogenes* to cleave both C3a and C5a (41) demonstrates a potential for this lethal pathogen to abrogate major components of the human neutrophil chemoattractant repertoire. We also determined that SpyCEP cleavage of CXCL8 abolished the ability of the chemokine to bind to heparin. These observations generated the hypothesis that interaction with GAGs might be central to activation of the receptors CXCR1 and CXCR2 by CXCL8. This is supported by our demonstration that the removal of neutrophil surface GAGs by glycanases can abolish the ability of freshly isolated neutrophils to respond to CXCL8, but leave responses to fMLF broadly intact. This is reminiscent of a previous report by Hoogewerf and colleagues (38) in which binding of [^125^I]-CXCL8 to CXCR1^+^ transfectants was significantly reduced following treatment with a mixture of glycanases. Similarly, treatment of CXCR1 transfectants with heparinase was shown by Wang and Richmond to result in a reduction in CXCL1 binding and receptor activation (42). Our findings are also keeping in with an earlier report in which significant reductions in neutrophil chemotaxis and receptor binding were observed with variants of CXCL8 truncated at positions 58 and 60 (23). In that study, the CXCL8 variants were chemically synthesized and refolded, although the effects of truncation on the correct folding of the chemokine were not monitored. In this study, we have described an immunology and clinically relevant truncation, which retains intact disulfide bridges within CXCL8 (13). Taken together, both studies indicate that the final 13 amino acids at the C terminus are critical for chemokine function.

**FIGURE 7.**
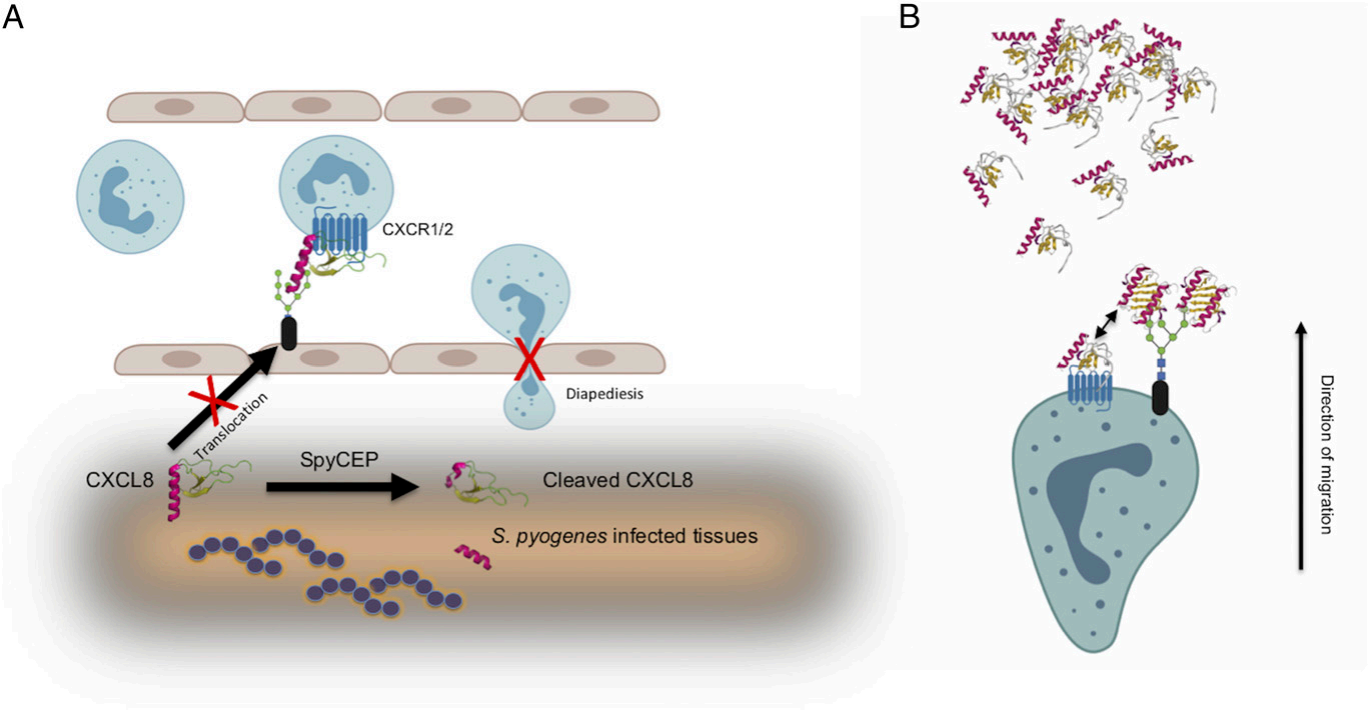
Loss of GAG binding following SpyCEP cleavage impairs CXCL8 translocation, presentation, and neutrophil chemotaxis. (**A**) shows how SpyCEP is envisaged to impair the initial recruitment of neutrophils by CXCL8. Loss of GAG binding following cleavage by SpyCEP results in chemokine that is unable to be translocated to the endothelial surface and subsequently be presented as a haptotactic gradient to direct neutrophil diapedesis. (**B**) shows the requirement for GAGs in neutrophil migration along a gradient of CXCL8 (direction indicated by the arrow). Cell surface GAGs encounter chemokine and bind it, increasing the local concentration of chemokine surrounding the neutrophil by assisting in chemokine oligomerization. Chemokine monomer can dissociate from the oligomer and activate the chemokine receptor (double-headed arrow).

Placing this information in the context of what we already know about chemokine:endothelial GAG interactions (37), the effects of SpyCEP cleavage upon CXCL8 biology, can be summarized as follows. First, GAG binding by the CXCL8 C terminus has been shown to be a prerequisite for translocation to the endothelium (40). Thus, CXCL8 generated in the tissues in response to invasive strains of *S. pyogenes* will be presumably rendered unable to be translocated to the luminal surface by SpyCEP cleavage, impeding presentation of the chemokine to neutrophils via the endothelium and subsequent neutrophil diapedesis (Fig. 7A). Second, the neutrophil glycocalyx sequestrates chemokines on the neutrophil surface (both monomer and higher order species such as dimers), in effect “sampling” the gradient at the leading edge of the migrating leukocyte. Previous studies have shown dimeric CXCL8 to have a higher affinity for GAGs than monomeric CXCL8 (43), but reduced efficacy and potency at CXCR1, in chemotaxis assays (44). Although both monomeric and dimeric CXCL8 bind GAGs, they are unable to bind to CXCR1 or CXCR2 while GAG bound (45). We therefore envisage that the neutrophil GAG serves to increase the local concentration of chemokine in the vicinity of the receptors (Fig. 7B). In the absence of the gradient sampling afforded by GAGs, migration along the chemokine gradient is much less efficient. As an analogy, consider an automobile taking a bend in the road after sunset. The beams from the headlights enable the driver to sample the environment and seeing the bend, steer the vehicle appropriately with negligible loss of speed. In contrast, in the absence of headlights and reduced environmental sampling, as the driver approaches the bend, it is necessary to slow down to reorientate the vehicle and safely navigate the bend, leading to less-fluent movement along the road. It is interesting to note that morphological differences in neutrophils navigating gradients of fMLF or CXCL8 have been reported previously by Yamauchi and colleagues. Notably, neutrophils migrating along an fMLF gradient displayed a fan-like, widely spread lamellipodium at the leading edge with a compact body and short tail. In contrast, neutrophils navigating a gradient of CXCL8 showed a more focused lamellipodium with a longer cell body and tail (46).

We conclude that GAGs on the surface of the neutrophil are an essential component of effective migratory responses to CXCL8 and that SpyCEP ruthlessly exploits this to render CXCL8 inactive in vivo (33). This raises several interesting questions. First, is sampling by GAGs required for efficient navigation of other chemokine gradients by neutrophils? This question requires additional experimentation to answer conclusively. There is perhaps a clue in the broad spectrum activity of SpyCEP for all ELR^+^ chemokines [(47) and Fig. 1B]; although, it could be that in evolving activity against a principal neutrophil chemoattractant in CXCL8, SpyCEP has “unwittingly” acquired the ability to degrade other neutrophil attractants. Second, are GAGs on the surface of other leukocytes critical for the navigation of chemokine gradients? Previous studies of monocytes (36) and T cells (48) have shown that glycanase treatment results in reduced intracellular signaling in response to the chemokines CXCL4 and CCL5, although these studies were not extended to analyses of cell migration. Finally, can we learn lessons from SpyCEP in terms of targeting the interactions of GAGs with chemokines for therapeutic benefits? Mutants of CXCL8, which are likely to bind to SpyCEP, but have reduced activity at CXCR1 and CXCR2, have been described by others and may be a useful starting point for drug discovery (49, 50). Provided such molecules clear the typical hurdles of bioavailability and target occupancy that have beset many small molecule chemokine receptor antagonists (51), they may present themselves as candidate SpyCEP inhibitors with potential for the adjuvant management of invasive group A streptococcal infections.

## Supplementary Material

Data Supplement
