# Supplementary material for: A Requirement for Neutrophil Glycosaminoglycans in Chemokine:Receptor Interactions Is Revealed by the Streptococcal Protease SpyCEP
Source: J Immunol. 2019 Apr 22;202(11):3246–55. doi: 10.4049/jimmunol.1801688 (PMC6526389; doi:10.4049/jimmunol.1801688)
Supplement: Data Supplement [file JI_1801688.zip › JI_1801688_Supplemental_Figures_1.pdf]

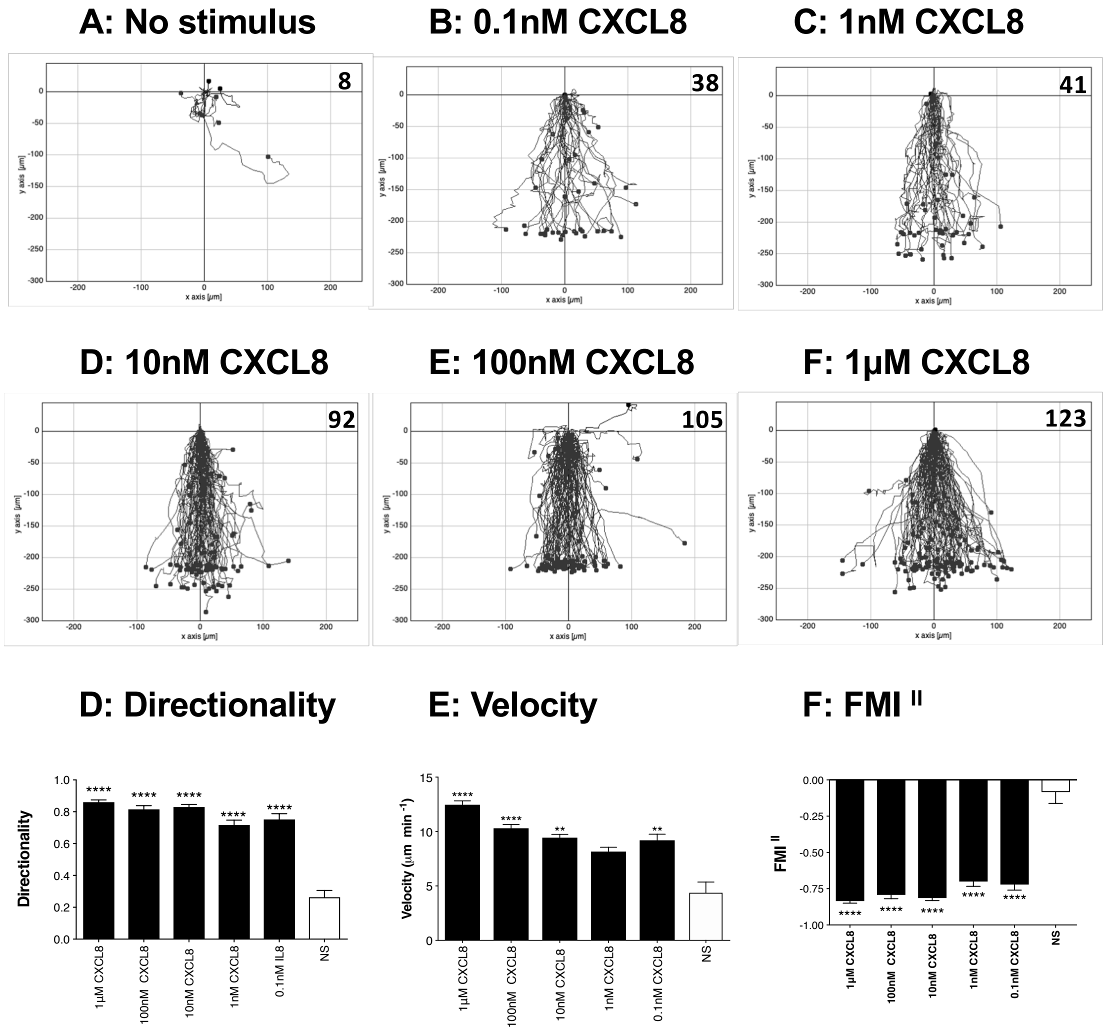

Supplementary Figure 1. Neutrophil migration along gradients of CXCL8 generated by the addition of different concentrations of chemokine.

Panels A-F show the collated tracks of individual migrating neutrophils from three independent experiments, using neutrophils isolated from three different donors. Numbers in the top right hand corner of each plot relate to the total number of cells tracked for each condition. Panels G-I show velocity, directionality and FMI$^{||}$ of the data in panels A-F following tracking analysis. Error bars represent the SEM. Statistically significant differences between various chemokine concentrations versus no stimulus (NS) were determined by one-way ANOVA with Tukey's' post-test (**= $p<0.01$), ****= $p<0.001$).

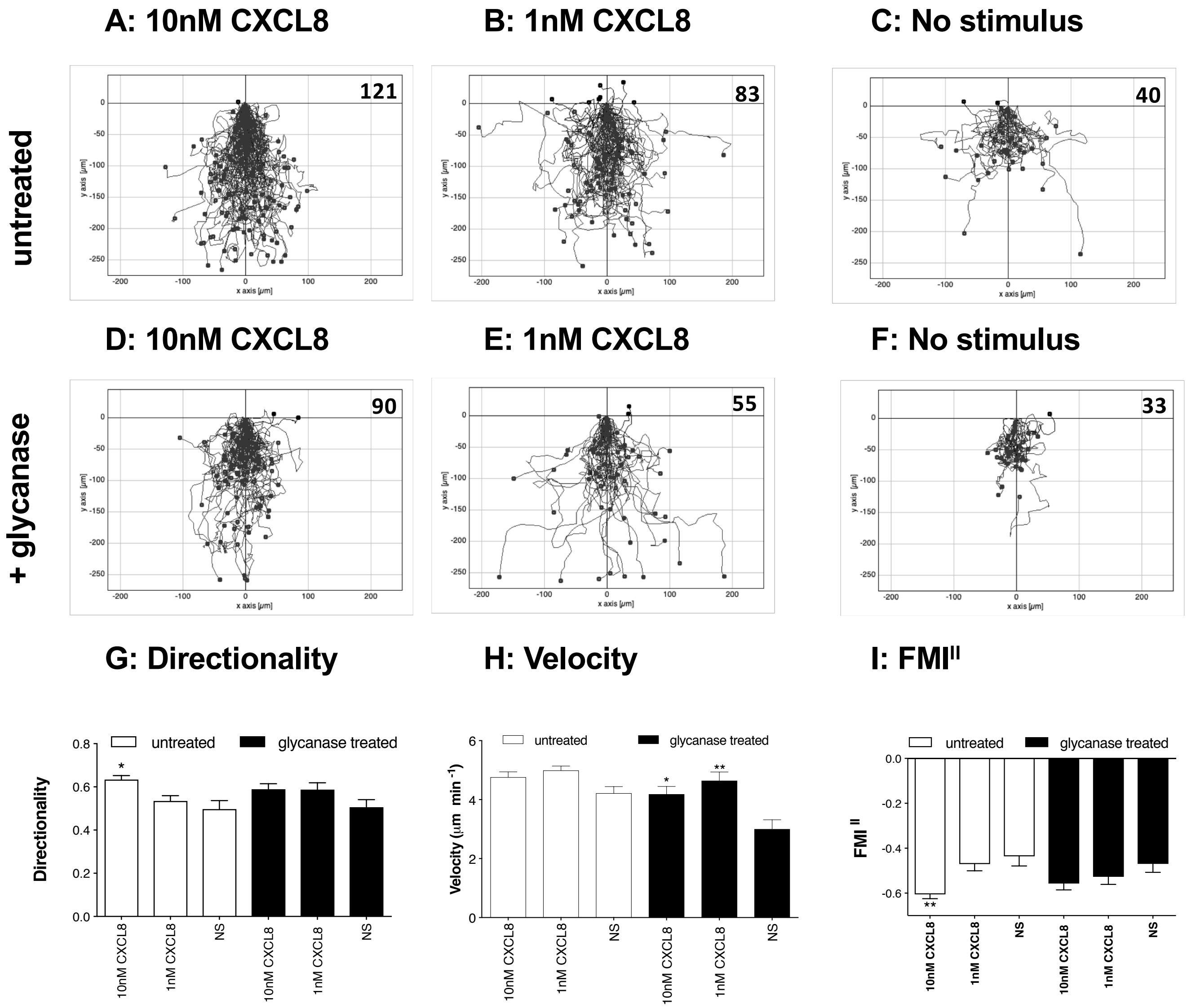

Supplementary Figure 2. Glycanase treatment impairs neutrophil migration along CXCL8 gradients of CXCL8 generated by the addition of different concentrations of chemokine.

Panels A-F show the collated tracks of individual migrating neutrophils (duplicate conditions) pooled from three experiments using different donors. In panels A-C, neutrophils were incubated in buffer alone prior to migration, while in panels D-F, cells were treated with a glycanase cocktail. Gradients of CXCL8 were established by the addition of 1µl 10nM CXCL8 and 1µl 1nM CXCL8 with the total number of tracked cells shown in the top right hand corner. Panels G-I show velocity, directionality and FMI$^{||}$ of the data in panels A-F following tracking analysis. Error bars represent the SEM. Statistical significances between responses to individual chemokine concentrations and the corresponding basal response (No Stimulus, NS) were determined by one-way ANOVA with Tukey's' post-test (* = $p<0.05$, **= $p<0.01$).
