# Supplementary material for: A Requirement for Neutrophil Glycosaminoglycans in Chemokine:Receptor Interactions Is Revealed by the Streptococcal Protease SpyCEP
Source: J Immunol. 2019 Apr 22;202(11):3246–55. doi: 10.4049/jimmunol.1801688 (PMC6526389; doi:10.4049/jimmunol.1801688)
Supplement: Data Supplement [file JI_1801688.zip › JI_1801688_Supplemental_Material_2.pdf]

Legends for Videos

Supplementary Video 1

Migration of freshly isolated human neutrophils along a gradient of intact CXCL8. Scale bar denotes 92 microns.

Supplementary Video 2

Migration of freshly isolated human neutrophils along a gradient of CXCL8 cleaved by SpyCEP. Scale bar denotes 92 microns.
